# Oxygen Consumption (V’O2) and physical Strainas measured by the occupational activity of cleaning personnel

**DOI:** 10.1186/s12995-018-0185-x

**Published:** 2018-01-19

**Authors:** M. J. Fröhlich, R. F. Kroidl, T. Welte

**Affiliations:** 1 Department of Respiratory Medicine, Medizinische Hochschule Hannover, Carl-Neuberg Str. 1, 30625 Hannover, Germany; 2 Stade, Germany

**Keywords:** Occupational workload, Real-life setting, Ergospirometry, Cardiopulmonary exercise testing, Disability

## Abstract

**Background:**

The aim of the study was to determine the physical effort and energy expenditure needed over a working period of 45–60 min, specifically for the occupational activity of cleaning. The effort was demonstrated in absolute terms (V’O2), in relation to the involved person’s maximum physical capacity (peak V’O2) and in relation to the individual aerobic-anaerobic threshold (V’O2 at VT1, the point when lactate starts to accumulate but can be cleared). In addition to this, the aim was to verify the suitability of portable ergospirometry in determining the occupational workload in a real-life setting.

**Methods:**

Thirty-five cleaners performed a bicycle ergospirometry to determine their maximum physical capacity (peak V’O2 = L/min) and their aerobic-anaerobic threshold (V’O2 at the Ventilatory Threshold 1 [VT1]). This was followed by portable ergospirometry lasting 45–60 min while pursuing regular cleaning activities.

**Results:**

Performance V’O2 (the average oxygen consumption over 45–60 min of work- time) was 1.06 L/min or 4.4 METs. This was scarcely lower than the individual V’O2 at VT1 and approached 45% of the maximum physical capacity (peak V’O2). In addition, there was positive feedback regarding the wearability of the portable device. The dropout rate was low.

**Conclusion:**

The occupational activity of cleaning was defined as a “committed activity”, performed close to the upper limit of the continuous physical capacity (approaching V’O2 at VT1). The positive feedback and a low dropout rate proved good acceptance of portable ergospirometry in this field of work over a 45–60 min period.

## Background

A frequent medical task involves the evaluation of an individual’s physical capacity with regard to his or her work demands. According to the recommendations of the WHO and its ICF-classification [1] such a judgment should include the total bio-psycho-social context of the individual. As part of the bio-psycho-social context the clinician needs to assess the physical capability of the person to perform his occupational activity. The performance demand (load) should be reflected against the performance ability (capacity), leading to the concept of “load to capacity”. Two questions must be answered:

- How great are the physical demands caused by the work?
- How high is this person’s physical capacity? It should be kept in mind that usual conditions and not peak performances are involved.

In Germany, physical workload is estimated according to the REFA-classification (REFA: Reichsausschuß für Arbeitszeitermittlung, now known as Verband für Arbeitsgestaltung, Betriebsorganisation und Unternehmensentwicklung e. V.). It has four gradings: “light”, “light to moderate”, “moderate” and “hard” [2]. This classification dates back to the 1920s and 1930s and primarily relates to carrying and holding work with emphasis placed on different physical positions.

In order to gain better and more concrete information as well as numeric values for performance demands, studies on energy consumption in diverse professional sectors were carried out in the 1950s and 1960s [3–6]. Oxygen uptake (V’O2 in L/min) was determined as the central measured parameter, providing energy consumption (in kcal) via the caloric equivalent. The measurements did not exclusively monitor conditions at the actual workplace (i.e. field tests) but were mostly carried out in standardised settings.

Energy expenditure is also the basis of the compendium of physical activities as compiled by Ainsworth et al. [7, 8]. This compendium takes the metabolic rate obtained during quiet sitting down (resting period) as the standard metabolic rate. It is assigned a valued with 1 MET (4184 kJ/kg body weight/h). The compendium lists 605 specific activities. The activities are classified according to the number of MET needed to perform them and classifies activities in terms of light, moderate or vigorous intensity activities. For example, “interior cleaning” is valued 3,01 MET. The compendium was compiled to standardize and classify the physical intensity of activities in previously done research. It is based on data acquired before the 1990’s, before the use of portable ergospirometry. It does not take individual variation (for example: fitness levels or medical disabilities) into account.

According to pertinent literature and widely accepted consent in occupational medicine, a person can be declared “fit for work” if the continuous workload does not exceed 40% of the individual’s peak V’O2. The aerobic-anaerobic threshold is regarded as the upper limit of the continuous physical performance capacity [9–14].

Given the possibilities of portable ergospirometry, we have a new and precise way to ascertain physical workload under authentic working conditions. It is important to demonstrate the workload in relation to the maximal capacity (“load to capacity”). Portable ergospirometry has been used in the field of occupational medicine. Among other fields it has been used in research pertaining to firefighters, in forestry and in the military [15–18]. Most of the common occupations have however not been investigated and not all studies were done in real-life settings (i.e. how does marching on an indoor treadmill relate to a field march in battle, or do firefighters experience the same amount of stress when performing a pre-trainer practise routine in comparison with a real firefight). In one recent study concerning municipal refuse collectors the “load to capacity” was evaluated by use of portable ergospirometry in a real-life setting [19]. The implementation of portable ergospirometry in daily practice remains an area of interest that needs to be evaluated further. Its use is not yet standard in determining the workload of an individual.

### Study goal

The goal of this study was firstly to determine in a group of cleaning personnel how great the physical performance needs to be to carry out cleaning work and how this activity is related to the actual maximum performance capacity and the aerobic-anaerobic threshold.

Secondly, our study focussed on determining if portable ergospirometry is suited for displaying the actual workload in a workplace related field test.

## Methods

### Participants and study design

Employees were recruited from the cleaning departments of rehabilitation centres on the island of Borkum as well as employees from several hotels and guest houses. All participants were of good health and gave informed consent before study entry. The study was presented to and approved by the ethical committee of the “Medizinische Hochschule Hannover”. Included were persons ranging in age from 18 to 65 years.

### Measurements

At first the maximum physical capacity (peak V’O2) was determined by an exhaustive bicycle ergospirometry. This was done in the facilities of the “Knappschaft-Bahn-See” rehabilitation centre. A Ganshorn Power Cube LF8, 5F paired with an Ergoline Ergometrics 900 ergometer in a semi-recumbent position was used. A Radiometer Copenhagen ABL700 was used to conduct capillary blood gas analyses.

**Fig. 1:**
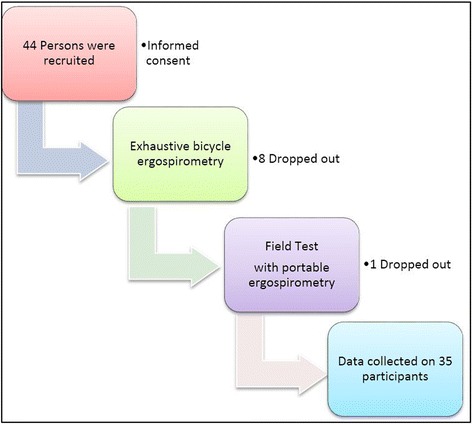
The Procedural Path

The exhaustive bicycle ergospirometries were carried out according to current clinical practise [20–22]. They were scheduled either early in the morning or early in the afternoon. The following test protocol was used: After 3 min of rest and 3 min of unloaded pedalling at 60 rpm, the workload was started at 25 watts. The workload was increased gradually in intervals of one minute. The increases were estimated according to the person’s prevailing physical abilities and with the help of tables, formulas and clinical experience in order to reach exhaustion after about 10 [8–15] min. Every subject had an individual testing protocol. Physical exhaustion was determined by clinical judgement, by use of the Borg Scale and by passing the aerobe-anaerobe threshold, V’O2 at VT1 (Ventilatory Threshold 1) [21], the point when lactate starts to accumulate but can be cleared. Capillary blood gas analyses were conducted at rest and close to exhaustion. The results were compiled in tables and depicted in the 9-panel plot according to Wasserman [20, 21]. The individual aerobic-anaerobic threshold was assessed predominantly with the use of panels 5, 6 and 9. As reference values, we used data from Wasserman and Jones [20]. All results were reviewed and validated by the second investigator. (Fig. 1).

**Fig. 2:**
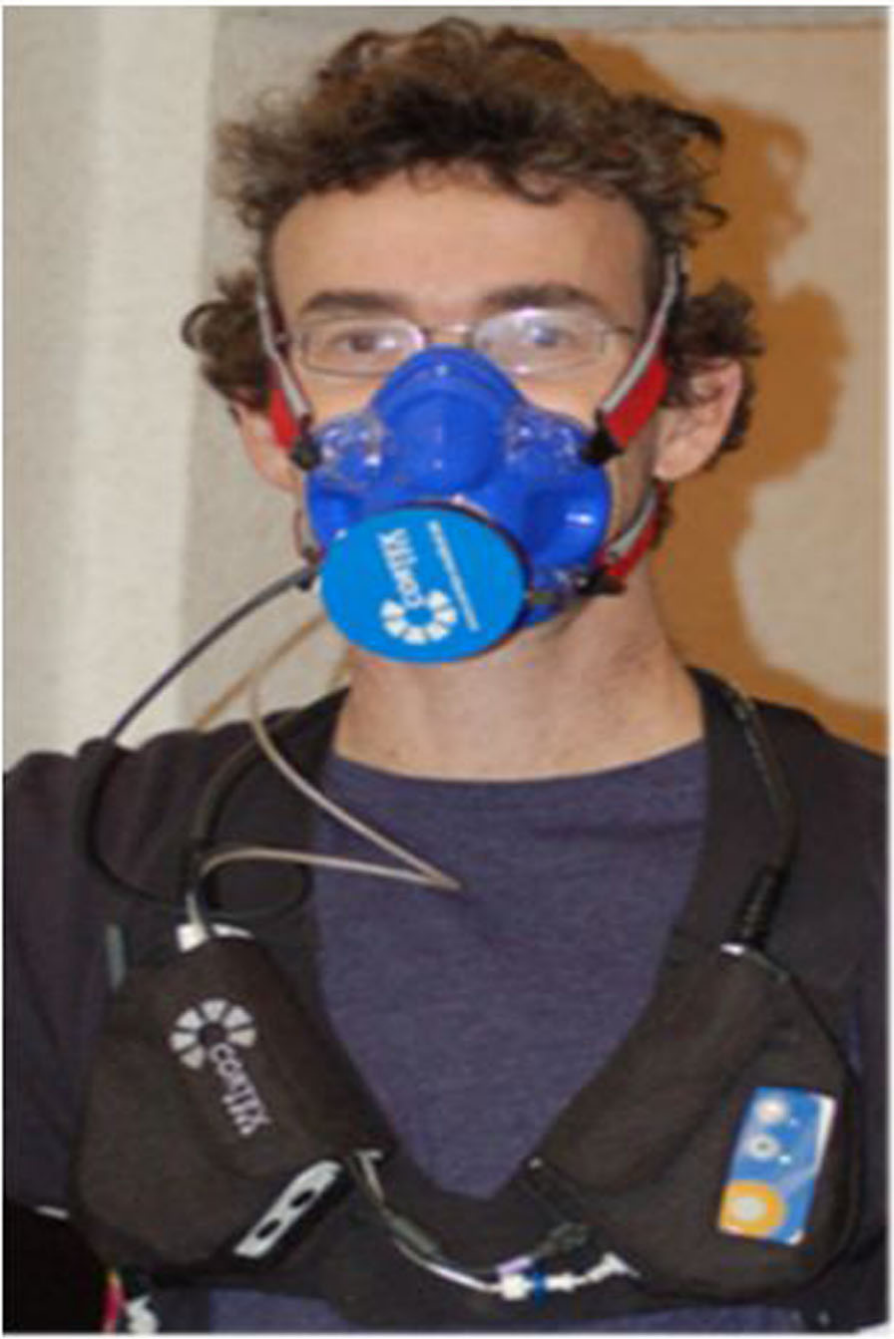
Test person wearing the portable ergospirometry device. Source: Own image library, reproduction with subjects’ permission

At a later stage, the tests were continued by using portable ergospirometry while the cleaners carried out their cleaning work, the “field test”. The cleaners were accompanied to their actual workplace and were monitored during their usual, daily work routine. After applying the portable ergospirometry device and measuring for 2–5 min at rest, the cleaners resumed their regular work. The cleaners were asked to continue the work that they were doing at their usual pace. The specific tasks were not standardized. The aim was to measure the workload in a real-life setting. The work consisted mostly of mopping and vacuuming floors, making beds and cleaning bedrooms as well as bathrooms. The aim was to record a period of between 45 and 60 min. The recording time variation was caused by work requirements. For example, recording continued until a subject had finished cleaning a specific section. (Fig. 2).

The Metamax 3B device® by Cortex, Leipzig, was used along with a pulsar pulse belt by Pulsar®. The Metamax 3B device® consists of two approximately 10 cm × 8 cm × 5 cm size parts which are connected by a cable. The cable is hung around the neck and the parts are attached with a harness to the front of the chest. The device itself weighs about 500 g. A mask, made of soft silicone, is placed with an elastic belt system over the face. A sensor piece fits onto the mask and connects to the chest device. The collected data can be stored in the device and/or sent wirelessly to a normal PC. The field test was only performed on persons who had previously undergone stationary ergospirometry without complications.

### Statistical analysis

We compiled and analysed the data with the use of Microsoft Excel 2013 (v15.0) and its statistical calculators (Microsoft Redmond campus, Redmond, Washington, United States). Categorical variables are shown as numbers (n) and percentages (%). Continuous variables are shown as mean ± SD, unless indicated otherwise. For comparisons a two-sided paired t-test was used as appropriate. All reported *p*-values are two-sided unless indicated otherwise; p-values < 0.05 were considered statistically significant.

## Results

Altogether 44 persons were recruited. Due to 9 dropouts (20%) a total of 35 subjects, 4 men (11%) and 31 women (89%) made up the test group. No prioritising in terms of sex was made. The mean age was 37.4 years (Standard Deviation, SD = 9.6) the median 37 years (Interquartile Range, IQR = 31–45). The mean height was 169 cm (SD 7.8), the median 168 cm (IQR = 163–172.5). The mean weight was 66 kg (SD = 18.3), the median 69 kg (IQR = 64–83). The mean BMI was 23.1 kg/m^2^ (SD = 5.8), the median 25.8 kg/m^2^ (IQR = 23.2–27.95). The mean recorded time in the field test was 52 min (SD 10.7), the median 50 min (IQR = 48–55).

It is noteworthy that the method of portable ergospirometry was well tolerated and accepted. Minor complaints such as increased sweating and oral dryness were reported in half of the cases. Work mobility was only slightly impaired. Altogether (stationary and portable ergospirometry combined) nine participants opted out. Reasons given were claustrophobia (four participants), the participant relocated (one), restriction of free movement during work (two participants) and revocation of consent without reason (two participants). The acceptance of these investigations by the working environment resulted in mixed responses: the appearance of an employee wearing a mask was (understandably) unusual and sometimes a cause for concern among bystanders. These concerns were, however, dealt with easily by the attending investigator.

The results showed that the resting V′O2 obtained during the bicycle ergospirometry compared well with the resting V′O2 measured before the field test.

- Bicycle ergospirometry: mean resting V′O2 0.38 L/min (SD = 0.11), median 0.35 L/min
  (IQR = 0.31–0.41)
- Portable ergospirometry: mean resting V′O2 0.38 L/min (SD = 0,12), median 0.41 L/min (IQR = 0.32–046)

A paired t-test revealed *p* = 0.19, stating no statistically relevant difference.

The mean peak V’O2 gained from bicycle ergospirometry was 2.06 L/min (SD = 0.54) or 28 ml/kg/min (SD = 7.4). The median peak V’O2 was 1.9 L/min (IQR = 1.78–2.16) or 26 ml/kg/min (IQR = 23–28). It corresponds to approximately 9 MET.

The mean V’O2 at VT1 (gained from bicycle ergospirometry) was 1.28 L/min (SD 0.34). Median V’O2 at VT1 was 1.25 L/min (IQR = 1–1.48).

To qualify the data, we introduced two terms:

- **Performance V’O2:** Total oxygen consumption while carrying out the work.
  This value includes the oxygen consumption at rest in addition to V’O2 allocated to the specific workload. The mean performance V’O2 was 1.06 L/min (SD = 0.24), the median performance V’O2 was 1.02 L/min (IQR = 0.92–1.19). This corresponds to approximately 4.5 MET.
- **Work V’O2:** Additional oxygen consumption needed to perform work, not including the oxygen consumption needed for basal metabolism. The mean work V’O2 was 0.68 L/min (SD = 0.19), the median work V’O2 was 0.65 L/min (IQR = 0.6–0.79). Therefore, the mean work-specific energy demand was calculated at (0.68 × 4.82) ≈ 3.3 kcal/min [21]. This is the energy expenditure needed for this specific professional activity.

Stationary semi-recumbent bicycle ergospirometry is not entirely suitable for determining the maximum physical capacity in relation to the measurements gained with a portable device. This is due to peak V’O2 being higher when weight-bearing activities are performed as opposed to non-weight-bearing activities [23–25]. We increased the peak V’O2 value as determined with the bicycle ergospirometry (non-weight-bearing activity) by 15% to 2.37 L/min in order to compare it with the “field test” of cleaning work (weight-bearing activity). The cleaner’s workload amounted to 45% of the maximum available physical capacity. Performance V’O2 reached 90% of the V’O2 at VT1.

**Fig. 3:**
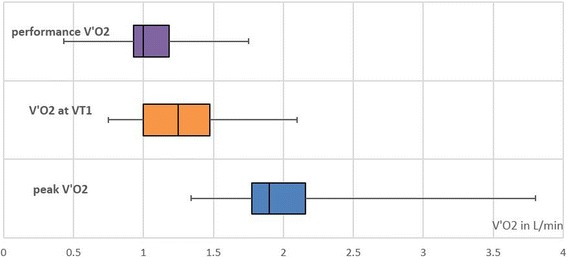
Comparison of performance V’O2, V’O2 at VT1 and the peak V’O2

A t-test between performance V’O2 and V’O2 at VT1 revealed *p* = 0.0015, confirming a statistically relevant difference. (Fig. 3).

## Discussion

The physical workload of cleaning work, especially how this activity relates to the individual’s actual maximum performance capacity and the aerobic-anaerobic threshold was noticeably higher than expected. The performance V’O2 almost reached 45% of the peak V’O2 and 90% of the V’O2 at VT1. According to pertinent literature and widely accepted consent in occupational medicine, a continuous permanent workload of around 40% of peak V’O2 is seen as the upper limit of continuous physical performance capacity [9–14]. Cleaning work exceeded this value, but not by a large margin. The work was done just within the boundary of predominantly aerobic metabolism.

If we use the criteria of the Compendium of Physical Activities by Ainsworth et al. [7, 8], our study showed that cleaning work could be classified as a moderate activity (3–6 MET) as it accounts for approximately 4.5 MET. Our study also showed the energy expenditure to be considerably higher than the assigned value of 3.01 MET (interior cleaning, 7, 8).

Work V’O2 is defined as the additional oxygen consumption needed to perform work, not including the oxygen consumption needed for the basal metabolism. This corresponds to a work-specific energy demand of approximately 3.3 kcal/min. This is relevant because if our results are compared with the studies completed during the 1950’s and 1960’s (Max Planck Institute, Dortmund) [3–6], we find that the results are very similar. With the technical possibilities available at that time, they measured the average energy consumption for cleaning work to be approximately 4 kcal/min.

Portable ergospirometry was suited for displaying the actual workload in field tests. The handling of the portable ergospirometry device turned out to be simple and intuitive. The measured values were plausible and the resting V′O2 compared well with that of the bicycle ergospirometry. The measurements were obtained without any complications. Mobility during the operation was marginally limited but restrictions were generally well tolerated. Claustrophobia seldom arose and portable ergospirometry was indeed practical in this setting. It is, however, important to mention that portable testing was only carried out on persons who had already tolerated face masks during earlier bicycle ergospirometry. Furthermore, not all occupations allow this type of testing, e.g. a salesperson or a telephone operator in a call centre cannot be tested. Also, it was noted that a cleaner wearing the portable ergospirometry device did (at times) elicit quite a bit of curiosity amongst bystanders. These factors could prove to be restricting in terms of the utilisation of portable ergospirometry testing in occupational medicine. Wearing the portable ergospirometry device (the mask) is not comfortable (sweating, dry mouth) and testing over longer periods of an entire shift, e.g. 4–6 or 8 h, is therefore not feasible.

The stationary bicycle ergospirometry in semi-recumbent position is not completely suitable for determining the maximum physical capacity in relation to the measurement done with the portable device. This is due to the difference in peak V’02 between weight-bearing and non-weight-bearing activities. Literature showed that a weight-bearing activity produces a higher peak V’O2 value of about 15% in comparison to a non-weight-bearing activity [23–25]. Having done the capacity measurements with an ergometer in semi-recumbent position, we corrected the peak V’O2 measurement obtained by means of bicycle ergospirometry through increasing it with 15% in order to compare it to the field test. The peak V’O2 measurement obtained by means of a bicycle ergospirometry as a surrogate for maximum V’O2 remains, however, a noteworthy limitation of our study.

## Conclusion

These real-life measurements showed that the performance requirements for professional cleaning work are higher than expected. The REFA-classification as traditionally used in Germany today would classify cleaning workload as “light work”. Our study shows that cleaning work reached the upper limit of continuous physical capacity. Thus, we do not classify cleaning as “light” work.

We propose the introduction of an extended classification system that would compare the prevailing physical capacity of a person with the real-life demands of the occupation, based on the concept of “load to capacity”. Our study showed that with the current increasing availability of stationary and portable ergospirometry the amount of physical effort needed to perform work and the physical capacity involved can be assessed, compared and studied further [26, 27]. In order to increase the accuracy we advise the use of a treadmill ergospirometry to obtain the maximal physical capacity.

In our opinion the present classifications do not provide an adequate representation for the workload of professional cleaning and therefore we suggest that a new term “committed activity” could be used to describe the work that was carried out at the upper limit of continuous physical capacity.

## Abbreviations

IQR: Interquartile Range
MET: Metabolic Equivalent of Task
Peak V’O2: The highest oxygen consumption achieved in a ergospirometry
Performance V’O2: Total oxygen consumption while performing the work
REFA: Reichsausschuss für Arbeitsstudien, currently: Verband für Arbeitsgestaltung, Betriebsorganisation und Unternehmensentwicklung e. V.
RER: Respiratory Exchange Rate
Resting V′O2: Oxygen consumption needed for the basal metabolism
SD: Standard deviation
V’O2 at the VT1: Ventilatory Threshold 1. The point where lactate starts to accumulate but can be cleared
V’O2: Oxygen consumption
Work V’O2: Additional oxygen consumption needed to perform work, not including the oxygen consumption needed for the basal metabolism
